# Strategies for low-molecular-weight heparin management in pregnant women with mechanical prosthetic heart valves: A nationwide survey of Dutch practice

**DOI:** 10.1016/j.ijcchd.2022.100373

**Published:** 2022-04-12

**Authors:** Marco Voortman, Jolien W. Roos, Jennichjen Slomp, Arie P.J. van Dijk, Berto J. Bouma, Gertjan T.J. Sieswerda, Philippine Kiès, Anna Boer, Willem M. Waskowsky, Clemens von Birgelen, Lodewijk J. Wagenaar

**Affiliations:** a Department of Cardiology, Thoraxcentrum Twente, Medisch Spectrum Twente, Enschede, the Netherlands; b Department of Cardiology, Erasmus Medical Centre, Rotterdam, the Netherlands; c Department of Clinical Chemistry, Medlon, Medisch Spectrum Twente, Enschede, the Netherlands; d Department of Cardiology, Radboud University Medical Center, Nijmegen, the Netherlands; e Heart Center, Department of Clinical and Experimental Cardiology, Amsterdam UMC, University of Amsterdam, Amsterdam Cardiovascular Sciences, Amsterdam, the Netherlands; f Department of Cardiology, University Medical Center Utrecht, Utrecht, the Netherlands; g Department of Cardiology, Leiden University Medical Centre, Leiden, the Netherlands; h Department of Cardiology, University Medical Center Groningen, Groningen, the Netherlands; i Department of Cardiology, Isala Heart Centre, Isala Hospital, Zwolle, the Netherlands; j Department of Health Technology and Services Research, Faculty of Behavioural Management and Social Sciences, Technical Medical Centre, University of Twente, Enschede, the Netherlands

**Keywords:** Pregnancy, Mechanical heart valve, Anticoagulation

## Abstract

**Background:**

In this study we investigated current Dutch practice of low molecular weight heparin (LMWH) treatment in pregnant women with mechanical prosthetic heart valves (MPHV) in order to evaluate how management can be optimized.

**Methods:**

Between December 2020 and February 2021, we conducted a survey among Dutch congenital cardiologists of tertiary centers in the Netherlands. We collected and analyzed written, unstructured, open questionnaires that were send to all 8 specialized pregnancy heart teams.

**Results:**

Response was obtained from all centers (response rate 100%). The preferred LMWHs were nadroparin (62.5%), dalteparin (25%), and enoxaparin (12.5%). After replacing vitamin K antagonist (VKA) with LMWH, 7 centers measured the first anti-Xa level within a week, and 1 center measured anti-Xa levels daily until targeted levels were reached. All centers monitored weekly peak anti-Xa levels (4–6 h post-dose) throughout pregnancy. Four out of 8 centers monitored additional trough (i.e. pre-dose) anti-Xa levels, and 3 of these 4 centers switched to LMWH 3 times daily to achieve target levels when necessary.

**Conclusions:**

In Dutch clinical practice, a considerable variation exists in LMWH management for pregnant women with MPHV. In some centers, LMWH was dosed 3 times daily to maintain target anti-Xa levels. Standardizing treatment strategies would allow systematic assessment in prospective studies.

## Abbreviations

**LMWH** =: low molecular weight heparin
**MPHV** =: mechanical prosthetic heart valve
**VKA** =: vitamin K antagonist
**ACHD =**: adult congenital heart disease

## 1 Introduction

Pregnant women with a mechanical prosthetic heart valve (MPHV) are at high risk of thrombo-embolic complications due to the hypercoagulable state of pregnancy combined with the inherently thrombogenic MPHV [1,2]. Furthermore, pregnant women with MPHV are at an increased risk of miscarriage, fetal death, and congenital abnormalities in the fetus when high doses of vitamin K antagonists (VKAs) are required [2,3]. To avoid these complications, replacing VKA with low molecular weight heparin (LMWH) may be considered between weeks 6 and 12 or throughout the entire pregnancy, as recommended in current guidelines [4,5]. However, in daily clinical practice effective and safe anticoagulation remains challenging.

Despite advancements of LMWH treatment by monitoring anti-Xa activity, LMWH is still associated with high rates of maternal complications [6,7], especially when administered improperly or monitored carelessly, or if patients are non-compliant [[8], [9], [10]].

Although previous studies reported that valve-thrombosis still occurs despite compliance with therapy [3,10], a recent study demonstrates that with proper administration and monitoring, LMWH can be used with a minimum of complications in this population [11]. A major issue still remains: target peak anti-Xa levels (4–6 h post-dose) at 0.8–1.2 U/mL, as recommended in current guidelines, are associated with subtherapeutic trough (i.e. pre-dose) anti-Xa levels, targeted at ≥0.6 U/mL [[11], [12], [13], [14]]. Although international guidelines provide general recommendations [4,5], monitoring regimens and dosage changes are generally left to the treating physicians or pregnancy heart teams.

In the current study, we investigated Dutch practice of LMWH treatment in pregnant women with MPHV by means of a survey among pregnancy heart teams in order to evaluate how management can be optimized.

## 2 Methods

A survey was designed based on current literature, guidelines recommendations and professional experience of the authors. The survey contained 8 questions, regarding treatment of pregnant women with MPHV, dose changes of LMWH and monitoring of anti-Xa levels (Appendix A).

The questionnaire was distributed on December 8, 2020 to members of the working group for Adult Congenital Heart Disease (ACHD) of the Netherlands Society of Cardiology. We targeted ACHD cardiologists representing a pregnancy heart team of an expert referral center.

The questionnaire consisted of open questions and respondents were asked for unstructured comments to reflect the local management of their pregnancy heart team. Our intention was to generate quantifiable ‘safety net’ data, which means that important new issues would not be missed by asking closed questions. This type of data will encourage attention to non-response bias and reliability of coding [15].

If the questionnaire was not returned after one month, a reminder was sent and if necessary the questionnaire was sent once more. The survey was closed on February 8, 2021. The survey was performed on a voluntary basis, and responses were anonymized for the analysis. In the tables, expert centers are presented in a randomly chosen order.

## 3 Results

The survey had a response rate of 8/8 (100%). Eight ACHD cardiologists from different centers filled in the questionnaire, representing all pregnancy heart teams of tertiary expert centers in the Netherlands: 6 academic teaching hospitals and 2 non-academic tertiary teaching hospitals.

### 3.1 Switching to low molecular weight heparin: start-up phase

**Fig. 1:**
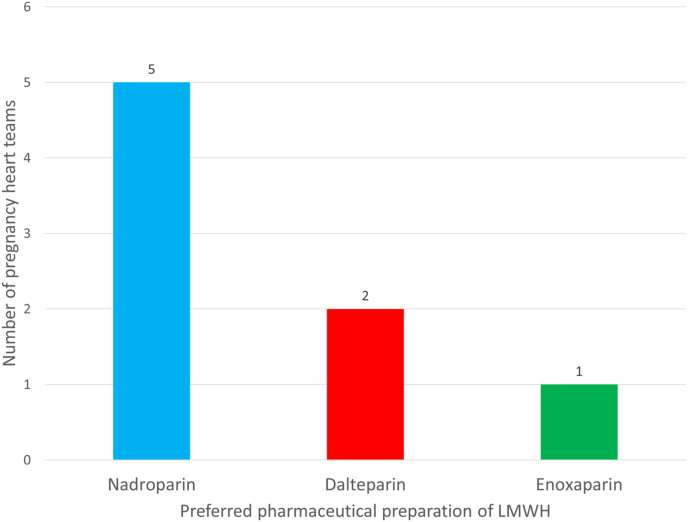
Different LMWHs preferred by Dutch pregnancy heart team for pregnant patients with MPHV. Abbreviations: LMWH = low molecular weight heparin; MPHV = mechanical prosthetic heart valve.

The different LMWHs preferred by pregnancy heart teams are shown in Fig. 1. The first choice of most centers was nadroparin (62.5%), while other centers preferred dalteparin (25%) or enoxaparin (12.5%). In all centers, the appropriate therapeutic doses of LMWH was based on total body weight. In the start-up phase, 1 center (12,5%) carried out daily measurement of anti-Xa levels until target levels were reached. In that center, patients were admitted to the hospital when the distance between a patients home and the hospital was considered too far away for commuting on a daily basis. In the other 7 centers (87.5%), the first anti-Xa level measurement was performed within one week after replacing VKA by LMWH.

### 3.2 Monitoring of anti-Xa levels during pregnancy

Table 1 demonstrates the different target anti-Xa levels that pregnancy heart teams used for monitoring. In 5 centers (62.5%) the peak target anti-Xa levels were 0.8–1.2 U/mL, in 2 centers (25%) the target anti-Xa levels were 0.7–1.2 U/mL, and in 1 center (12.5%) the target anti-Xa level was 0.7–1.5 U/mL. In 2 centers, higher peak target anti-Xa levels of 1.0–1.2 U/mL were used in pregnant patients with MPHV in mitral position or right-sided positions. In 4 centers (50%), monitoring was based on trough anti-Xa levels: in 3 centers the target anti-Xa level was ≥0.6 U/mL, and in 1 center it was ≥0.4 U/mL.

**Table 1:** Target anti-Xa levels used by Dutch pregnancy heart teams.

No.	Peak anti-Xa level (U/mL)	Trough anti-Xa level (U/mL)	Peak anti-Xa level (mitral/right sided valves) (U/mL)
1	0.7–1.2		
2	0.8–1.2	>0.6	1.0–1.2
3	0.7–1.2		
4	0.8–1.2		
5	0.8–1.2		1.0–1.2
6	0.8–1.2	>0.4	
7	0.7–1.5	>0.6	
8	0.8–1.2	>0.6	

Target anti-Xa levels used in different Dutch pregnancy heart teams. The order of centers is shown randomly. Abbreviations: LMWH = low molecular weight heparin.

### 3.3 Dose adjustments during pregnancy: stable phase

In the stable phase, when target anti-Xa levels were reached, weekly monitoring of anti-Xa was performed in all centers. Four of all 8 centers specified that additional monitoring was performed after dose adjustment of LMWH for out-of-range anti-Xa levels. In all centers, the anti-Xa levels could be analyzed by a laboratory within 1–4 h and dose adjustments were made on the same day. Table 2 features strategies for dose adjustment to maintain anti-Xa levels within the target range. Six of the 8 centers formulated local practice guidelines for changing LMWH doses. If anti-Xa levels were too low, 3 centers administered LMWH 3 times daily instead of 2 times daily; these 3 centers were all centers that measured trough levels. One center responded that dosage was increased in steps of 0.1 ml 2-times-daily, until the target level was reached.

**Table 2:** Strategies for dose adjustment to maintain anti-Xa levels by Dutch pregnancy heart teams.

No.	Dose changes during pregnancy in women with MPHV
1	No specific dose adjustment strategy
2	Initially increase the dose of LMWH 2 times daily. If that is no longer possible, LMWH is used 3 times daily.
3	No specific dose adjustment strategy
4	Increase the dose of LMWH 2 times daily
5	Never once a day. Increase the dose of LMWH 2 times daily. Also “learn to inject” beforehand. When in doubt about skills of administration, observe self-administration
6	Increase frequency to LMWH 3 times a day, if the targeted levels are much too low. Otherwise increase LMWH 2 times a day to 0.8 ml.
7	Initially increase the frequency to LMWH 3 times daily. If insufficient effect, then increase the dose of LMWH 3 times daily
8	Increase the dose of LMWH 2 times daily in steps of 0.1 ml

Strategies for dose changes to maintain anti-Xa levels in pregnant women with MPHV. The order of centers is shown randomly. Abbreviations: LMWH = low molecular weight heparin; MPHV = mechanical prosthetic heart valve.

## 4 Discussion

The use of optimal strategies for LMWH treatment in pregnant women with a MPHV is regarded as an important matter. Yet, among Dutch pregnancy heart teams we found considerable variation in the clinical practice of LMWH treatment and monitoring. All centers monitored weekly peak anti-Xa levels (i.e. 4–6 h post-dose) throughout pregnancy. Half of the interviewed pregnancy heart teams performed additional monitoring based on trough anti-Xa levels (i.e. pre-dose), and most of these centers endorsed a protocol that allowed increasing the frequency of LMWH application to three times daily in order to maintain targeted anti-Xa levels.

The main goal of anticoagulation therapy in pregnant women with MPHV is to prevent valve thrombosis and its lethal consequences for both mother and fetus. Although vitamin K antagonists (VKAs) are considered the most effective regimen for the prevention of pregnancy-related thromboembolic complications, they are associated with a significant risk of fetal complications, including embryopathy and miscarriage [[1], [2], [3]]. Therefore, in the majority of pregnant women, VKA are (temporally or throughout pregnancy) replaced by LMWH, which does not cross the placenta and hence does not have any direct effect on the fetus [[1], [2], [3],^16^]. Due to predictable pharmacokinetics and pharmacodynamics, it is generally not considered necessary to routinely monitor LMWH efficacy. However, during pregnancy, fixed dosing of LMWH appears to be associated with unacceptably high maternal morbidity and mortality [[8], [9], [10]]. Consequently, in this setting measurement of anti-Xa activity is performed to guide dosing of LMWH therapy. Yet, in pregnant women with MPHV both target anti-Xa level and optimal monitoring schedule are still subjects of debate.

A number of studies have demonstrated that monitoring peak anti-Xa levels, as recommended in current guidelines, does not reliably guide anticoagulation efficacy [9,[11], [12], [13], [14],^16^]. Despite adequate target peak anti-Xa levels, pregnant women with MPHV still may develop valve thrombosis [9,16], and recent studies suggest the importance of adding routine measurements and maintenance of trough anti-Xa levels in order to assure adequate anticoagulation [[11], [12], [13], [14]]. Nevertheless, based on current literature, it cannot be determined whether monitoring anti-Xa activity in pregnant women with MPHV improves maternal outcome [6,7].

The results of our study illustrate that although Dutch pregnancy heart teams comply with the general recommendations of international guidelines (Table 3)*,* there is a wide variation in LMWH treatment of pregnant women with MPHV [4,5]. In particular, target levels and the frequency of monitoring were inconsistent among centers, and variations could reflect both non-uniformity between guidelines and the limited data that are available to support the association between target anti-Xa monitoring and clinical efficacy. The European guidelines provide more specific recommendations, yet based on expert opinion. In particular, it is recommended to implement changes to LMWH regimen during pregnancy in an in-hospital setting, with daily anti-Xa level measurements until the target is reached. In addition, higher peak anti-Xa levels of 1.0–1.2 U/mL should be targeted for mitral and right-sided prosthetic valves [5], while the US guidelines do not state such specific recommendations [4].

**Table 3:** Current clinical practice guideline recommendations for LMWH management in pregnant women with MPHV.

Guideline	American College of Cardiology/American Heart Association (ACC/AHA) [4]	European Society of Cardiology (ESC) [5]
	Patients with Valvular Heart Disease 2020	Guidelines for the Management of Cardiovascular Diseases during Pregnancy 2020
LMWH recommendations in pregnant women with MHV	For pregnant women with mechanical prostheses who require a warfarin dose >5 mg/d to achieve a therapeutic INR, dose-adjusted LMWH (with a target anti-Xa level of 0.8–1.2 U/mL at 4–6 h after dose) at least 2 times per day for all 3 trimesters may be considered (2a B-NR)	Discontinuation of VKAs between week 6 and 12, and replacement with adjusted dose intravenous UHF or adjusted dose LMWH twice daily, should be considered in patients with a warfarin dose >5 mg/day
	For pregnant women with mechanical prostheses who require >5 mg/d of warfarin to achieve a therapeutic INR, dose-adjusted LMWH (with a target anti-Xa level of 0.8–1.2 U/mL at 4–6 h after dose) at least 2 times per day during the first trimester, followed by warfarin during the second and third trimesters, is reasonable (2b B-NR)	During the second and third trimesters, LMWH with anti-Xa level monitoring and dose adjustment may be considered in women who need a high dose of VKAs after patient information and consent. (IIb)
Dosage recommendations for LMWH	Dose-adjusted LMWH should be given at least 2 times per day, with close monitoring of anti-Xa levels.	Starting dose for LMWH is 1 mg/kg body weight for enoxaparin and 100 U/kg for dalteparin, twice daily subcutaneously.
Monitoring	Effective dose monitoring includes weekly measurements of anti–factor Xa levels, with additional monitoring after dose adjustment	In-hospital change to LMW, with daily anti-Xa levels until target. Then weekly when the target anti-Xa level is achieved.
		It is recommended to implement changes in the anticoagulation regimen during pregnancy in hospital. (IC)
	For pregnant women with mechanical prostheses, LMWH should not be administered unless anti-Xa levels are monitored 4–6 h after administration and dose is adjusted according to levels (3 B-NR)	LMWH is not recommended when weekly anti-Xa level monitoring and dose-adjustment is not available. (IIIC)
Target peak anti-Xa levels	Target to Xa level of 0.8–1.2 U/mL, 4–6 h after dose.	In pregnant women with LMWH, it is recommended to target anti-Xa levels 4–6 h post-dose at 0.8–1.2 U/mL (aortic valve prosthesis) or 1.0–1.2 U/mL (mitral and right-sided valve prostheses). (IC)
Target trough anti-Xa levels	Measurement of trough levels to maintain a trough Xa level >0.6 U/mL may help women maintain therapeutic anticoagulation while on LMWH	In pregnant women with LMWH, in addition to monitoring peak anti-Xa levels, monitoring trough levels targeted at > 0.6 U/mL may be considered (IIb C)

Abbreviations: LMWH = low molecular weight heparin; VKA = vitamin K antagonist.

We found that half of the Dutch pregnancy heart teams measure additional trough anti-Xa levels. Similar findings were reported by Vause et al. [16], a population-based study that described the practice of anticoagulation in women with MPHV in the United Kingdom. Monitoring regimens of LMWH were provided for 32 of 58 women, showing a substantial variation in target ranges and frequency of anti-Xa level measurement with a median number of 10 (range 0–75) measurements. Besides monitoring peak target levels, additional trough levels were measured in 52% of women. Furthermore, a recent study by Dos Santos et al. [17] compared anticoagulation management of two obstetric cardiac centers and observed a substantial variation in anticoagulation monitoring, with one center monitoring monthly anti-Xa activity. In addition, they also reported inconsistencies in peri-partum anticoagulation strategies and mode of delivery.

This study reports that a majority of Dutch pregnancy heart teams preferred nadroparin. At this moment, no LMWH is officially approved (i.e. labelled) for pregnant women with mechanical valves. Nevertheless, in clinical practice a variety of pharmaceutical preparations, such as nadroparin, dalteparin, and enoxaparin, are administrated in this patient population for the prevention of thromboembolic complications. Although these LMWHs are used for the same indications, they differ in physicochemical and pharmacokinetic characteristics [18,19]. Collignon et al. [19] compared the pharmacokinetic profiles of three LMWH in healthy volunteers and found that the apparent total body clearance of enoxaparin is significantly lower than that of nadroparin and dalteparin; and hence the apparent elimination half-life value differs among LMWHs (dalteparin: 2.8 h; nadroparin: 3.7 h; enoxaparin: 4.1 h). In the absence of randomized clinical trials comparing different LMWHs in pregnant women with MPHV, it is unclear whether these differences have any impact on clinical outcome and the incidence of thromboembolic complications. Yet, based on previous studies that demonstrated enoxaparin and dalteparin are well-tolerated, treating physicians or pregnancy heart teams might consider choosing one of these LMWH in this setting [10,11,23,24].

During pregnancy, the dose of LMWH that is required to maintain therapeutic levels of anticoagulation may increase. Barbour et all [12] showed that during pregnancy 85% of women with MPHV require an increase in dose. Hence, strict monitoring and good compliance are considered mandatory to prevent anticoagulant “failure” with occurrence of a valve thrombosis. Up to now, no specific recommendations for dose changes during pregnancy have been published, and there is a lack of robust dosing information to ensure adequate anticoagulation. In the current study, we found that in centers that only monitor peak anti-Xa levels, an upward dose adjustment was performed with two LMWH doses daily in order to achieve target anti-Xa levels. In contrast to that, the majority of centers which monitored both trough and peak anti-Xa levels considered switching to an intensified regimen based on LMWH 3 times daily. We believe centers measuring trough levels may feel more compelled to using new strategies in order to maintain target anti-Xa levels, including LMWH administration 3 times daily. This was also suggested in a recent study by Goland et al. [11] which reported that dose-adjusted LMWH therapy, aimed to achieve current guideline-recommended peak levels of anti-Xa, is associated with insufficient trough anti-Xa levels in 80% of the patient. Furthermore, it should be noted that in 31% of patients even with excessively high peak anti-Xa levels (>1.2 U/mL) adequate trough levels were still not achieved.

Our findings may be of clinical relevance in pregnant women with MPHV who cannot maintain adequate trough target levels, especially patients in whom peak levels are already excessively high. A limitation of using LMWHs for anticoagulation during pregnancy is their renal clearance. Due to far-reaching pregnancy-related changes in maternal physiology with an increased renal clearance and a higher volume of distribution, the anticoagulant effect of LMWH 2 times daily can in some patients be insufficient to maintain adequate trough and peak anti-Xa levels [25,26]. To the best of our knowledge, no published study –neither in pregnant nor in non-pregnant patients – has assessed the anticoagulant effect of LMWH 3 times daily. However, as in pregnant women apparent elimination half-life values of LMWH are shorter, it is fair to assume that in some patients LMWH 3 times daily may be required to achieve an uninterrupted, adequate levels of anticoagulation [[20], [21], [22]].

### 4.1 Limitations

Although the present survey was exclusively conducted in Dutch centers, we feel that the results are of global interest, as the findings can be compared to local clinical practice and may stimulate discussions about optimal dosing of LMWH and monitoring of anti-Xa levels. Nevertheless, several study limitations should be acknowledged. We received all information from experienced ACHD cardiologists, but we did not collect data on the specific characteristics of the individual respondents and the tertiary centers they are employed at. The questionnaire consisted of open questions, stated without clinical context or case description. This might have resulted in rather generalized answers that render the detection of subtle management differences in specific clinical scenarios difficult.

## 5 Conclusion and recommendations

Among Dutch pregnancy heart teams, there was considerable variation in LMWH management for pregnant women with MPHV, but their practice complied with the general recommendations of current guidelines. Based on pharmacokinetic studies of LMWH and knowledge about changes in maternal physiology during pregnancy, it is likely that in some pregnant women the anticoagulant effect of LMWH administered *2 times daily* can be insufficient, which may trigger a switch to dosing it *3 times daily*. We found that pregnancy heart teams that measured not only *peak* levels but also *trough* levels were more compelled to dose LMWH 3 times daily in order to maintain target anti-Xa levels. Standardizing LMWH treatment strategies for these rare high-risk pregnancies is desirable, as this would allow systematic assessment in prospective studies.

## Source of funding

This research did not receive any specific grant from funding agencies in the public, commercial, or not-for-profit sectors.

## Declaration of competing interest

The authors declare that they have no known competing financial interests or personal relationships that could have appeared to influence the work reported in this paper.

## Appendix A Questionnaire distributed to members of the committee for Adult Congenital Heart Disease of the Netherlands Society of Cardiology

1. Which low molecular weight heparin is 1st choice?
2. Which starting dose is used?
3. How often is the anti-Xa determined in the start-up phase?
4. Are only peak anti-Xa levels measured, only trough anti-Xa levels measured or both determined?
5. How soon will the results be known, and can the dosage be adjusted if necessary?
6. Which reference values of the anti-Xa are used?
7. Which dose change strategy is used (e.g. if the anti-Xa dose is too low, increase the dose or the same dose more often per day)?
8. How often is the anti-Xa monitored after the start-up phase?
